# Editorial: Socio-technical ecologies: Design for human-machine systems

**DOI:** 10.3389/frobt.2022.1037454

**Published:** 2022-11-25

**Authors:** Jean Botev, Ada Diaconescu, Heiko Hamann, Stephen Marsh, Francisco J. Rodríguez Lera

**Affiliations:** ^1^ VR/AR Lab, Department of Computer Science, University of Luxembourg, Esch-sur-Alzette, Luxembourg; ^2^ Information Processing and Communication Laboratory, Computer Science and Networks Department, Telecom Paris, Institut Polytechnique de Paris, Paris, France; ^3^ Institute of Computer Engineering, University of Lübeck, Lübeck, Germany; ^4^ Faculty of Business and Information Technology, Ontario Tech University, Oshawa, ON, Canada; ^5^ Robotics Group, University of León, León, Spain

**Keywords:** human-AI interaction, human-robot interaction, systems engineering, socio-technical design, collaboration

Computer system design has been driven predominantly by technical aspects and considerations. However, as computing systems are increasingly embedded and integrated into the world, they have an extended impact on individuals and societies. In this context, social and behavioral dynamics become equally essential components of computing systems research, notably including robotics and artificial intelligence. The increasing shift towards socio-technical systems and the integration of collaborative and cooperative aspects require new multidisciplinary efforts and a focus on the larger system context and evolution. In hybrid systems, i.e., artificial agents, robots, and humans interacting with each other, explicitly considering the underlying social relations and dynamics is integral to the ability to design robust, adaptive, purpose- and useful systems. Recent advances in machine learning and other computational techniques allow for the effective real-time analysis of complex interaction behavior. This holds both for the individual constituents as well as for the integration of subsystems. This highly multidisciplinary Research Topic, therefore, spans various journals including Frontiers in Psychology and Frontiers in Computer Science with their *Human-Media Interaction* sections, Frontiers in Robotics and AI with its Human-Robot Interaction and Multi-Robot Systems sections, and Frontiers in Artificial Intelligence with its AI for Human Learning and Behavior Change section.

Taking a systemic, cross-disciplinary perspective on human-AI and human-robot interaction, as well as their effects on the larger system context, we encouraged submissions that integrate dedicated findings on socio-technical settings from, e.g., neuroscience, psychology, sociology, economics, and other relevant disciplines, into novel interaction and system design approaches. Original research, reviews, tools, databases, benchmarks, and evaluation methods relative to the following topics were welcome.

The international submissions reflect this variety, with the group of accepted manuscripts comprising original research, methods, and review articles covering various perspectives on designing for human-machine systems that form socio-technical ecologies.

In their article, von Terzi et al. address the problem from a psychological perspective, comparing the fulfillment of needs while experiencing technologies and how that is linked to social context, i.e., private and public settings. The results indicate significant effects for relatedness and popularity, potentially improving systems design.

Raymond et al. analyze the problem of fairness in decentralized conflict resolution and how privacy limitations constitute a trade-off for fairness losses. A series of randomized and application examples compare different strategies and evaluate perspective/scope as the objective global fairness measure against the perceived local fairness.

Bagheri et al. look at transparent interaction-based learning mechanisms for human-robot collaboration to improve the training efficiency, efficacy, and performance of collaborative robots (cobots) working with non-expert human partners.

Leichtmann et al. review possible replicability issues in human-robot interaction and provide methodological, statistical, and systemic suggestions to harden and improve future studies regarding comparability, reliability, and robustness.

Finally, Boos et al. introduce a compliance-reactance model to evaluate new human-robot interaction approaches on the system level through behavioral and affective factors. Their framework translates well-known elements from inter-human social interaction to human-robot and other systems, underscoring the importance of providing social cues to successful deployment and social acceptance.

Other submissions covered topics ranging from user perception of privacy, over abstract UI concepts, to questions of presence in collaborative work and communication.

As human-machine systems are deployed and becoming a reality in numerous scenarios involving different stakeholders, there is a need to shift system design towards more holistic approaches, which consider second-order effects on the host environment (“habitat”) and, in consequence, on the embedded systems. This explicitly includes long periods of time where systems of systems form ecologies and co-evolve after deployment. Highly interactive settings, such as an advanced smart city scenario, comprise many heterogeneous systems, e.g., robots and/or artificial agents, crowd-sourced data, social media, location-based applications, swarms of delivery drones, cleaning or gardening robots. The goal is to improve urban life, such as transport systems, healthcare, infrastructure, and services, by collecting and analyzing data from a wide range of sensors and applications. However, many of these systems fail to adapt or serve their intended purpose within the larger socio-technical ecosystem as they are oblivious to the complex dynamics in their immediate context, let alone the effects on cities as larger organisms. It is therefore vital to establish an integral way of designing human-machine systems that form socio-technical ecologies, allowing them to respect and adapt to the ever-evolving context in which they are embedded.

We believe this Research Topic constitutes a step towards a more comprehensive, multidisciplinary view of human-machine systems and their design. Our thanks go to all reviewers for their in-depth assessments and to the authors for their contributions.

